# Sleep-like behavior and 24-h rhythm disruption in the Tc1 mouse model of Down syndrome

**DOI:** 10.1111/gbb.12198

**Published:** 2015-02-16

**Authors:** I Heise, S P Fisher, G T Banks, S Wells, S N Peirson, R G Foster, P M Nolan

**Affiliations:** † Harwell Science and Innovation Campus, MRC HarwellHarwell, UK; ‡ Nuffield Department of Clinical Neurosciences (Nuffield Laboratory of Ophthalmology), John Radcliffe Hospital, University of OxfordOxford, UK

**Keywords:** Circadian wheel-running, Down syndrome, sleep, Tc1, trans-species aneuploid mouse line

## Abstract

Down syndrome is a common disorder associated with intellectual disability in humans. Among a variety of severe health problems, patients with Down syndrome exhibit disrupted sleep and abnormal 24-h rest/activity patterns. The transchromosomic mouse model of Down syndrome, Tc1, is a trans-species mouse model for Down syndrome, carrying most of human chromosome 21 in addition to the normal complement of mouse chromosomes and expresses many of the phenotypes characteristic of Down syndrome. To date, however, sleep and circadian rhythms have not been characterized in Tc1 mice. Using both circadian wheel-running analysis and video-based sleep scoring, we showed that these mice exhibited fragmented patterns of sleep-like behaviour during the light phase of a 12:12-h light/dark (LD) cycle with an extended period of continuous wakefulness at the beginning of the dark phase. Moreover, an acute light pulse during night-time was less effective in inducing sleep-like behaviour in Tc1 animals than in wild-type controls. In wheel-running analysis, free running in constant light (LL) or constant darkness (DD) showed no changes in the circadian period of Tc1 animals although they did express subtle behavioural differences including a reduction in total distance travelled on the wheel and differences in the acrophase of activity in LD and in DD. Our data confirm that Tc1 mice express sleep-related phenotypes that are comparable with those seen in Down syndrome patients with moderate disruptions in rest/activity patterns and hyperactive episodes, while circadian period under constant lighting conditions is essentially unaffected.

Down syndrome is, with an incidence of about 1 in 700 live births, the most common autosomal aneuploidy in humans. The partial or whole triplication of the human chromosome 21 (Hsa21) (Lejeune *et al.* 1959) is associated with numerous features including congenital heart defects, gastrointestinal anomalies, craniofacial alterations, early-onset dementia and mental disturbances (reviewed by Malt *et al.* 2013). Among the brain deficits experienced by Down syndrome patients, increased sleep fragmentation is a consistent feature (Churchill *et al.* 2012; Diomedi *et al.* 1999; Fernandez & Edgin 2013; Levanon *et al.* 1999). Currently, there are numerous mouse models available partially reflecting the human syndrome. All are based on the conserved synteny between Hsa21 and mouse chromosomes 16 (Mmu16), Mmu17 and Mmu10 (Cole *et al.* 1998; Pletcher *et al.* 2001; Yu *et al.* 2010). Based on different regions of synteny, these mice reflect different aspects of the human condition and provide valuable models for further study. In assessing alterations in rhythmic activity and sleep–wake in mouse models, the majority of studies have focused on the Ts65Dn line containing approximately 60% of the mouse orthologues of human genes on Hsa21. Confoundingly, however, this mouse line is also trisomic for regions unrelated to Hsa21, including about 60 genes from centromeric Mmu17 (Duchon *et al.* 2011; Reinholdt *et al.* 2011). The behavioural outcomes of Ts65Dn studies are variable (Escorihuela *et al.* 1995; Martinez-Cue *et al.* 2002; Martinez-Cue *et al.* 2013; Reeves *et al.* 1995; Stewart *et al.* 2007).

The most novel and complete mouse model for Down syndrome is the Tc1 (transchromosomic, Tc(Hsa21)1TybEmcf) line (O'Doherty *et al.* 2005). This is a trans-species aneuploid line expressing a large portion of Hsa21 (83%, 269 genes) as a third copy. Several Down syndrome-related phenotypes have been detected in the Tc1 line including learning and memory deficits (Morice *et al.* 2008; O'Doherty *et al.* 2005), increased stereotypic grooming and activity in the open field (Galante *et al.* 2009), impaired balance and coordination on a static rod and impairments in motor skill learning on an accelerating Rotarod (Galante *et al.* 2009).

Given that Tc1 mice express many of the characteristic features of Down syndrome, we were prompted to determine their circadian activity and sleep-related parameters. Our approach has been to use two independent non-invasive methodologies. First, we used conventional wheel-running screens to monitor circadian locomotor activity under several lighting conditions. Second, a non-invasive video monitoring approach, estimating sleep based on pre-defined periods of immobility, was utilized (Fisher *et al.* 2012; Pack *et al.* 2007). This dual methodological approach allows one to assess multiple specific components of behaviour. These include estimates of circadian period, tau (*τ*), under constant conditions and wheel-running performance using the conventional wheel-running approach. Furthermore, the video-tracking method provides information about the timing, duration and consolidation of immobility-based sleep assessment and activity. By assessing animals in both tests, we have been able to detect significant latencies in Tc1 animals for light-induced activity suppression while rest–wake patterns in these animals are significantly disrupted reflecting the sleep-related findings in Down syndrome patients (Gigli *et al.* 1987; Levanon *et al.* 1999).

## Materials and methods

### Animals

Tc1 mice and wild-type littermate controls were bred at the Mary Lyon Centre, Harwell, and tested between 8 and 12 weeks of age; a total number of 31 mice were used in this study. The colony was maintained as an F1 (C57BL/6Jx129S8) colony, with a stable transmission frequency of more than 40% of progeny inheriting Hsa21 from their mothers. Owing to the loss of transmission of Hsa21, this mouse line cannot be kept on a pure genetic background (O'Doherty *et al.* 2005). DNA for genotyping was extracted from ear biopsies using 100 µl of 50 mm NaOH at 95°C for 90 min and buffered with 10 µl of 1 m Tris pH 7.5. Hsa21 present in Tc1 mice was identified by PCR using primers D21S55F (5′-GGT TTG AGG GAA CAC AAA GCT TAA CTC CCA-3′) and D21S55R (5′-ACA GAG CTA CAG CCT CTG ACA CTA TGA ACT-3′) specific to Hsa21 and control primers for myosin (MyoF: 5′-TTA CGT CCA TCG TGG ACA GCA T-3′ and MyoR: 5′-TGG GCT GGG TGT TAG TCT TAT-3′) resulting in PCR products of 208 and 245 bp, respectively.

All animal experiments were carried out under the guidance issued by the Medical Research Council in ‘Responsibility in the Use of Animals for Medical Research’ (July 1993) and Home Office Project Licence (No. 30/2686) and in accordance with the Animal (Scientific Procedures) Act 1986, UK. All experiments conformed to international guidelines on the ethical use of animals.

### Circadian wheel-running

Ten adult Tc1 male mice and ten littermate control males were singly housed in cages equipped with running wheels with food and water available *ad libitum* in light tight chambers with ambient temperature kept at 21 ± 2°C and 45–65% humidity (Banks & Nolan 2011). Mice were entrained under a standard 12:12-h LD cycle with the onset of light at *zeitgeber* time (ZT) 0 and dark onset at ZT12. After 8 days under light/dark (LD) conditions, animals were transferred to free-running conditions for 12 days in constant darkness (DD) followed by 14 days in constant light (LL). Wheel-running data were recorded and analysed using ClockLab (Actimetrics, Wilmette, IL, USA) using default settings to calculate all parameters measured. anova tests (SPSS, IBM, Armonk, NY, USA) were performed to identify differences between experimental groups.

### Video-tracking

Five adult Tc1 male mice and six littermate control males were singly housed in video-monitored standard home cages placed in light tight chambers with food and water available *ad libitum*. Video-tracking and sleep estimation were performed as described previously (Fisher *et al.* 2012) using a validated methodology which showed a correlation coefficient of more than 94% when compared with electroencephalography (EEG) recordings. This correlation has been shown not only for baseline conditions but also been confirmed following administration of sedatives (Zolpidem) or stimulants (caffeine) in a dose-dependent manner (Fisher *et al.* 2012). Mice were first kept under a standard 12:12-h LD cycle with at least a 72-h habituation period to the home cage prior to any recordings. After baseline data collection for a single 24-h LD cycle, a 3-h acute light pulse (LP) was presented during the dark period at ZT16 and data recorded for the duration of the LP and for 2-h segments immediately before and after the LP. Finally, animals were transferred to DD and data recorded over a full circadian cycle.

Videos were recorded at 12.5 frames per second (FPS) and saved in AVI format. Stored videos were analysed using ANYmaze software (Stoelting, Wood Dale, IL, USA) by tracking the centre of the animal with an immobility detection rate of 95%, a validated setting (Fisher *et al.* 2012) to prevent detection of movement caused by breathing during sleep. According to this validation, animals were recorded as asleep when immobile for more than 40 seconds. Data for LD or constant conditions were analysed in hourly bins, whereas data for the LP study (including pre- and post-LP) were analysed in 10 min bins to detect rapid changes in sleep–wake behaviour following acute changes in lighting conditions. Time spent immobile (asleep) is displayed as a percentage of the total time in a particular bin (1 h or 10 min). For example, if an animal is immobile for an entire 1-h bin, then immobility is scored as 100%. Immobility in this context is subsequently referred to as asleep. Furthermore, in order to facilitate comparison with wheel-running data, the *y*-axes for immobility were inverted so that higher percentages of immobility, representing estimated periods of sleep, correspond to lower *y*-axis values. Lower percentages of immobility, representing active periods, are displayed as higher *y*-axis values. Statistical analysis was performed using two-way anovas in SPSS (IBM).

## Results

**Figure 1:**
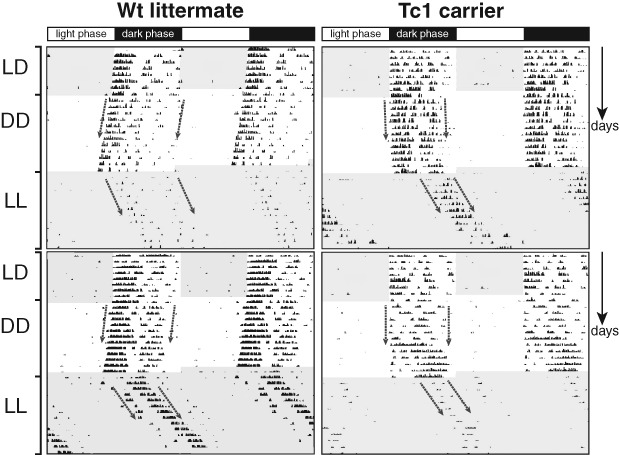
Double plotted actogram for Tc1 animals and littermate controls. Actogram showing wheel-running data in 12:12 light/dark (LD) for 8 days, constant darkness (DD) for 12 days, and constant light (LL) conditions for 14 days for two Tc1 carriers and two littermate controls. An acute LP for some of the animals was given at ZT 14 on the third night.

**Figure 2:**
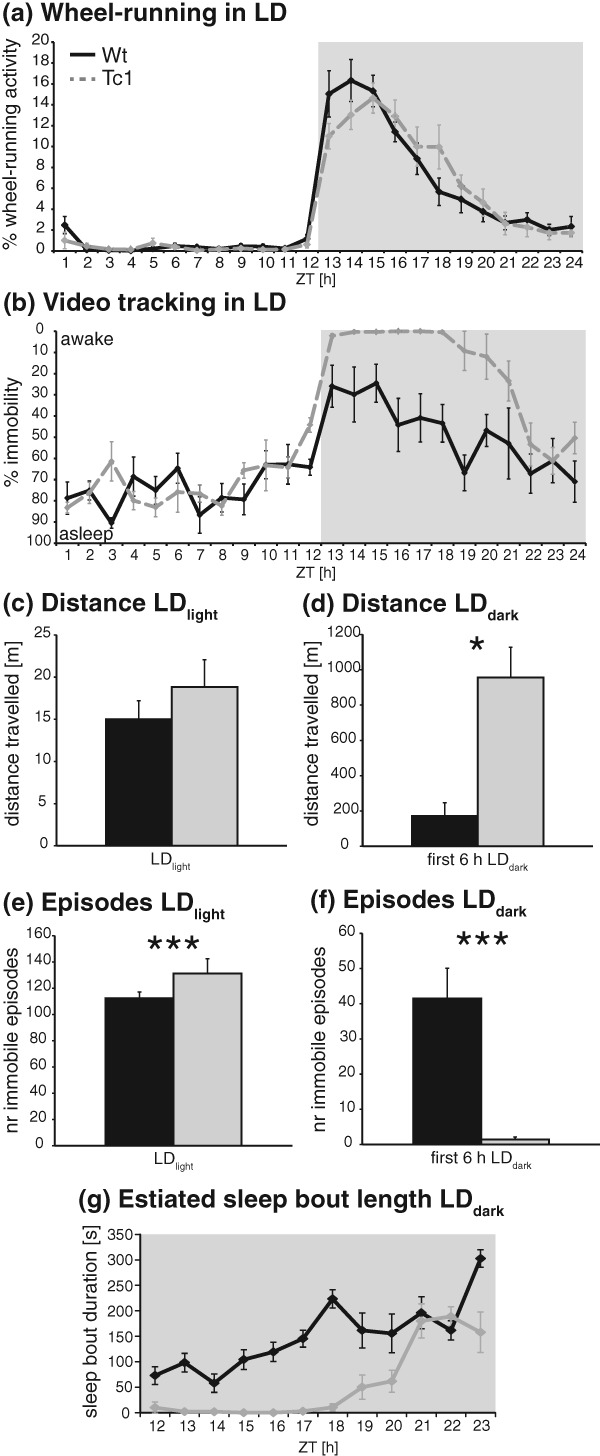
Activities of Tc1 and control mice under LD conditions. (a) Wheel-running: percentage of activity for wild-type (black) and Tc1 (grey) animals during 12:12-h light/dark (LD) conditions. (b) Video-tracking: percentage of immobility for wild-type and Tc1 animals during LD. For some data points, all Tc1 animals showed 0% immobility. (c–f) Video-tracking. (c) Total distance travelled during full 12 h of the light period. (d) Total distance travelled during first 6 h of the dark period. (e) Number of immobile episodes during full 12 h of the light period. (f) Number of immobile episodes during first 6 h of the dark period. (g) Estimated average sleep bout lengths during 12 h of the dark period. Averages were calculated per hourly bin. *P < 0.05; **P < 0.005; ***P < 0.002.

Using a conventional circadian wheel-running recording system, mice of both genotypes, Tc1 carrier mice and wild-type littermate controls, showed normal photoentrainment under a 12:12 LD cycle, with 90% of wheel-running activity occurring in the nocturnal phase and similar alpha (Table1, Figs. 1 and 2a). Comparison of wheel-running activity showed a trend towards a delay in the acrophase of wheel-running activity at the beginning of the dark phase for Tc1 animals, although this was not significant (Fig. 2a, wild-type acrophase at 22.00 ± 0.41 h and Tc1 at 20.96 ± 0.25 h, *F*_1,17_ = 1.553, *P* = 0.230). Furthermore, Tc1 animals ran significantly less during the dark phase of the LD cycle compared with their wild-type littermate controls resulting in lower average rotations per night (Table1). Remarkably, however, video-tracking of Tc1 animals showed a 6-h period sustained wakefulness (0% immobility), with the onset of the dark phase. Inspection of videos during this time confirmed that mutant animals conduct all of the behaviours expected, including locomotor activity, vertical activity, climbing, feeding and grooming, more frequently than wild-type littermate controls. Controls were asleep for approximately 20% of the time with sleep increasing at later stages in the dark phase (Fig. 2b). Tc1 animals were not only more mobile than littermate controls but more active with greater distances travelled (*F*_1,54_ = 100.91; *P* < 0.001; Fig. 2c). Inspection of video recordings during this period of hyperactivity showed that the number of immobile episodes was significantly lower for Tc1 animals (*F*_1,54_ = 45.88; *P* < 0.001), which is consistent with a constant and uninterrupted wakefulness (Fig. 2e). Average estimated sleep bout length was also significantly shorter (Fig. 2g, *F*_1,10_ = 0.842, *P* = 0.0218). In the light phase of the LD cycle, no significant differences in distance travelled and percentage of immobility could be observed (Fig. 2). However, the number of immobile episodes during the light phase was significantly higher for Tc1 animals compared with littermate controls (*F*_1,108_ = 5.72; *P* = 0.018), while average estimated sleep bout length was not significantly different (*F*_1,10_ = 7.367, *P* = 0.38), which is indicative of greater sleep disruption/fragmentation in triploid animals (Fig. 2f).

**Table 1:** Wheel-running parameters in Tc1 and control mice under three separate lighting conditions

	Wt	Tc1
Mean revolutions LD (± SEM)	2871 ± 789	2078 ± 430^*^
Nocturnal activity LD [%] (± SEM)	94.65 ± 1.5	96.27 ± 1.1
Amplitude LD (± SEM)	926 ± 95	824 ± 43
Alpha length LD (± SEM)	8.89 ± 0.38	8.10 ± 0.41
Mean revolutions DD (± SEM)	2956 ± 782	3106 ± 491
Tau DD (± SEM)	23.74 ± 0.05	23.79 ± 0.09
Amplitude DD (± SEM)	1183 ± 231	1137 ± 124
Alpha length DD (± SEM)	11.06 ± 0.49	9.26 ± 0.50^*^
Mean revolutions LL (± SEM)	1624 ± 417	1626 ± 744
Tau LL (± SEM)	24.83 ± 0.11	25.08 ± 0.10
Amplitude LL (± SEM)	869 ± 171	986 ± 187
Alpha length LL	9.01 ± 0.64	5.86 ± 0.50^*^

Average wheel-running revolutions under light/dark (LD) conditions and percentage of nocturnal wheel-running activity, amplitude and alpha in Tc1 and littermate controls (Wt). Mean wheel-running revolutions, Tau, amplitude and alpha during constant darkness (DD) and constant light (LL).

Asterisk (^*^) indicates a significant difference between genotypes (*P* < 0.05).

**Figure 3:**
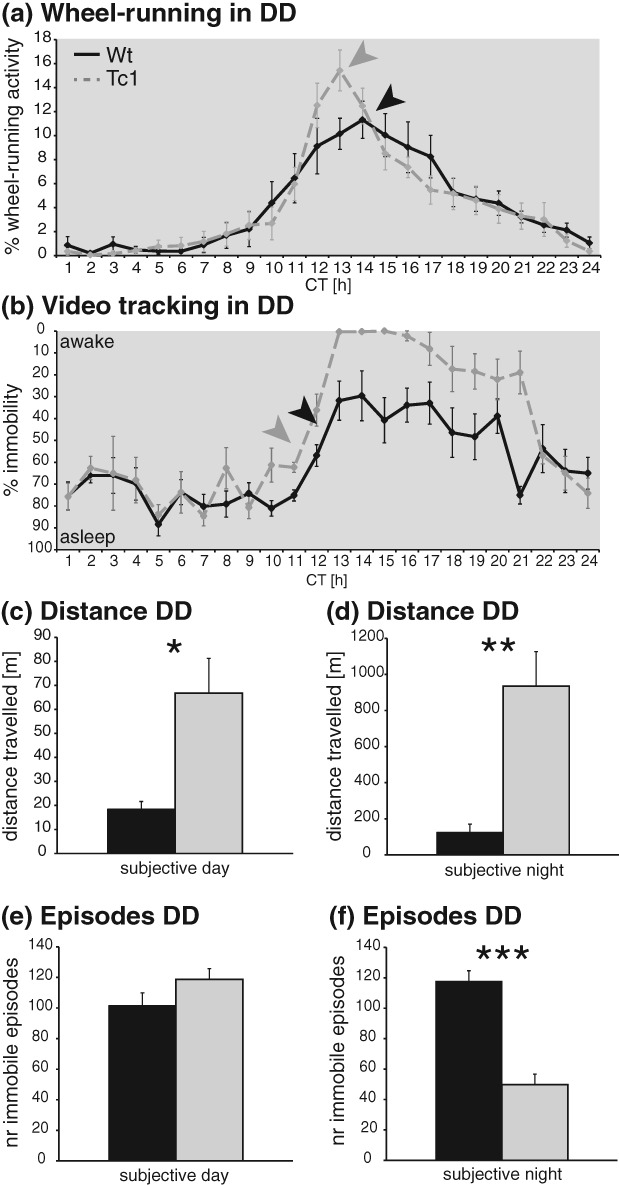
Activities of Tc1 and control mice under DD conditions. (a) Wheel-running: percentage of activity for wild-type (black) and Tc1 (grey) animals during constant darkness (DD). (b) Video-tracking: percentage of immobility for wild-type and Tc1 animals during DD. For some data points, all Tc1 animals showed 0% immobility. (c–f) Video-tracking. (c) Distance travelled during subjective day. (d) Distance travelled during subjective night. (e) Number of immobile episodes during subjective day. (f) Number of immobile episodes during subjective night. *P < 0.05; **P < 0.005; ***P < 0.002.

Under free-running conditions in DD, no significant differences in wheel-running amplitude or in average revolutions were identified. Neither was the circadian period significantly different between genotypes, but Tc1 animals displayed shorter alpha compared with wild-type littermates (Table1). When investigated in DD, the acrophase of wheel-running activity again showed a non-significant advanced trend for Tc1 carriers with a more defined and narrow peak of activity in comparison to wild-type littermate controls (Fig. 3a, *F*_1,18_ = 0.726, *P* = 0.405). In LL conditions, the internal period lengthened for all animals as expected. Average *τ*_LL_, revolutions, and amplitude were not significantly different for wild-type and Tc1 animals, but alpha was shorter for Tc1 animals compared with that for littermate controls (Table1).

The results of the video-tracking in DD were similar to those described for LD conditions. At the beginning of the subjective night, Tc1 mice maintained their state of sustained wakefulness (displayed as 0% immobility) compared with littermate controls, although the length of this period was shortened to 3 h of intense activity. This increased wakefulness in Tc1 animals shows a non-significant advanced trend of 1–2 h relative to that of wild types (latency to first immobile episode in subjective night, wild-type 67.94 min, Tc1 185.40 min, *F*_1,9_ = 1.338, *P* = 0.277). Wild-type littermates exhibited a level of immobility of about 30% at this time, again decreasing towards the end of the subjective night (Fig. 3b). Distance travelled by Tc1 animals was significantly higher not only during subjective night (*F*_1,108_ = 58.09; *P* < 0.001, Fig. 3d) but also during the subjective day (*F*_1,108_ = 10.02; *P* = 0.002, Fig. 3c). The latter effect is likely related to the advanced phase of activity seen in Tc1 animals. Finally, by comparing the number of immobile episodes in DD conditions, no significant differences between genotypes were detected during the subjective day whereas during the subjective night mutant animals exhibited a significantly lower number of immobile episodes (*F*_1,108_ = 44.68; *P* < 0.001, Fig. 3f).

**Figure 4:**
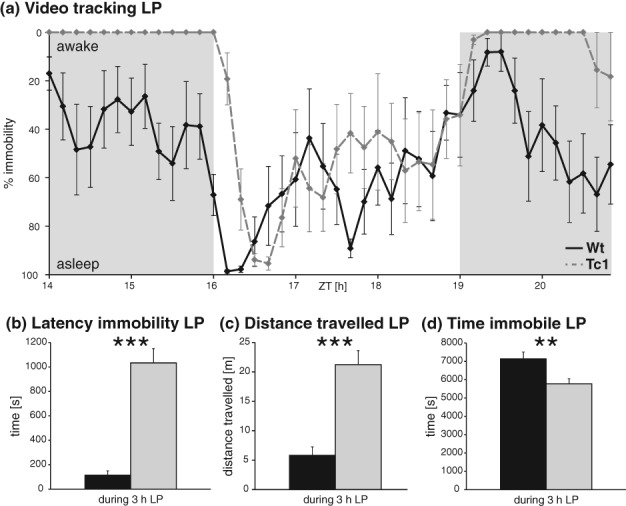
Activity suppression in Tc1 and control mice following a 3-h light pulse. (a–d) Video-tracking. (a) Percentage of immobility for wild-type (black) and Tc1 (grey) animals before, during and after the light pulse (LP, unshaded segment of graph). For some data points, all Tc1 animals showed 0% immobility. (b) Latency for first immobile episode during LP. (c) Distance travelled during LP. (d) Time immobile during LP. *P < 0.05; **P < 0.005; ***P < 0.002.

Introduction of an acute LP in the dark phase of the LD cycle should rapidly induce sleep in mice (Lupi *et al.* 2008; Muindi *et al.* 2013). Light-induced immobility-defined sleep was less pronounced for Tc1 animals during wheel-running (not significant, Fig. S1, Supporting information) and significantly delayed by around 20 min in Tc1 animals compared with that in their wild-type littermate controls in the video-tracking approach (Fig. 4a). This was confirmed as a significantly longer latency to the first occurrence of immobility-defined sleep (*F*_1,9_ = 66.17; *P* < 0.001, Fig. 4b). Analysis of the total amount of immobility-defined sleep and activity across the duration of the LP showed significant differences between genotypes with Tc1 animals travelling a greater distance (*F*_1,9_ = 33.40; *P* < 0.001, Fig. 4c) and spending less time asleep (*F*_1,9_ = 7.92; *P* = 0.020, Fig. 4d). Furthermore, comparing immobility between control littermates and Tc1 mutants in pre- and post-LP periods (darkness) showed much higher levels of activity for the Tc1 carrier group (Fig. 4a). This was consistent with the results of the 24-h LD and DD recordings (Figs. 3).

## Discussion

Using two diverse and complementary methodologies, conventional circadian wheel-running analysis and a novel validated video-tracking system that defines sleep based upon periods of immobility (Fisher *et al.* 2012), we have been able to gain new insights into the general activity, circadian function and sleep-related behaviours of the Tc1 mutant mouse line. Estimating periods of sleep based on immobility is a useful approach to assess sleep/wake behaviour in mice as it is a non-invasive technique that is both faster and more flexible for high-throughput analysis. It is also much more appropriate for mouse lines like Tc1, which show deficits in skilled motor function (Galante *et al.* 2009), a compromised health status and reduced survival rates after surgery. Moreover, as video-tracking results are so closely correlated with EEG data (Fisher *et al.* 2012), this approach has significant advantages over the use of more invasive tethered or telemetric EEG recording approaches as mutant animals may be unduly affected by anaesthesia and surgical procedures. In contrast, however, video-based assessment of sleep-like behaviour may be confounded by motor-skills differences in mutant mice and this possibility should be considered in studies such as this.

The use of multiple tests in this study has allowed us to measure disturbances in general activity and motor function. Wheel-running is a non-invasive test that has been used traditionally to measure circadian activity as well as phasic responses to LD conditions. The majority of studies suggest no alterations in circadian locomotor activity for Ts65Dn animals during a 12:12-h LD cycle (Martinez-Cue *et al.* 2002; Reeves *et al.* 1995) or in constant lighting conditions (Ruby *et al.* 2010). Surprisingly, however, Ts65Dn mice exhibit a hyperactive phenotype visible predominantly in the early hours of the dark phase that is not evident during the light phase (Escorihuela *et al.* 1995; Martinez-Cue *et al.* 2013). Moreover, a single independent study found that Ts65Dn animals exhibit a significant advanced phase in activity onset of approximately 4 h compared with wild-type mice under a 12:12-h LD cycle (Stewart *et al.* 2007). Overall, we detected only very subtle circadian phase-associated disturbances in Tc1 animals using this behavioural assessment. However, we did detect a reduction in the average wheel-running activity of Tc1 mutant mice during the dark phase of the 12:12-h LD cycle. Motor disabilities are a common symptom in Down syndrome patients (Spano *et al.* 1999). In earlier studies, Tc1 mice were shown to have impairments in skilled motor functions although general movement, gait, grip strength and other simple motor functions were unaffected (Galante *et al.* 2009), and this is reflected in our study where mice are more active while engaging in less wheel-running activity. This distinction is not unprecedented as we have shown in an earlier study using principal component analysis that wheel-running performance is independent of locomotor activity (Mandillo *et al.* 2014). Of course, we cannot discount the fact that Tc1 animals were less motivated to run on wheels. Additional studies testing animal motivation would need to be carried out to comment on whether this might contribute to the low wheel-running we found.

In contrast to the wheel-running study, Tc1 mice express a hyperlocomotor activity in the home cage as assessed using video-tracking. Hyperlocomotion had been previously recorded in Tc1 mice when they were assessed over short intervals in the open field test (Galante *et al.* 2009), but this is the first instance where consistent levels of hyperactivity have been recorded over long intervals in the home cage. This suggests that increased activity in Tc1 animals is not only precipitated by introducing mutant animals to a novel environment, as in the open field, but is expressed as an unprovoked behaviour in the familiar surroundings of the home cage. Galante *et al.* (2009) suggest that hyperactivity may be associated with a deficit in hippocampal function in Tc1 animals. Interestingly, impulsivity and hyperactivity are frequently reported for Down syndrome patients (Ekstein *et al.* 2011).

Our data also highlight the influence of LD phases on Tc1 activity disturbances. Hyperactivity in Tc1 animals is most evident at night, while the duration of this hyperactive phase is shortened when they are maintained in DD. Similar observations have been noted for Ts65Dn animals. For example, activity levels have been significantly higher during the dark phase in LD compared with those in DD or LL for Ts65Dn animals when compared with wild types in an actimetry study (Ruby *et al.* 2010). Also, Ts65Dn mice tested under white and red light conditions in an open field arena only showed a significantly increased number of line crossings under white light conditions when compared with control animals (Escorihuela *et al.* 1995). Light at night-time also highlights significant differences between Tc1 animals and littermate controls. Analysis of the LP data emphasizes how changing environmental lighting conditions can modulate the behaviour of Tc1 animals as evidenced in the delayed onset and reduction of light-induced sleep for the duration of the LP.

Down syndrome patients suffer from sleep disturbances such as increased daytime sleepiness, prolonged sleep latency at night, reduced amount and number of bouts of rapid eye movement (REM) sleep and sleep fragmentation (Carter *et al.* 2009; Diomedi *et al.* 1999; Grubar *et al.* 1986; Hamaguchi *et al.* 1989; Levanon *et al.* 1999). Sleep apnoea is thought to play a major role in causing sleep abnormalities in Down syndrome patients (Marcus *et al.* 1991) but is not the only cause (Levanon *et al.* 1999). In developing mouse models for Down syndrome, it is important that these sleep disturbances can be reflected accurately. Circadian and sleep/wake-related behaviour in the Ts65Dn mouse model shows similarities to Down syndrome patients with clear disturbances in EEG parameters showing decreased non-rapid eye movement (NREM) sleep and NREM bout durations associated with increased wakefulness during the light phase (Colas *et al.* 2008). Although not accompanied by EEG recordings, Tc1 animals exhibit a lower percentage of estimated sleep and delays in the onset of light-induced sleep in our study. Nevertheless, traits like sleep fragmentation and longer latencies for sleep onset in patients (Breslin *et al.* 2011; Carter *et al.* 2009) are reflected in delayed latency for light-induced sleep (LP) and a higher amount of immobile episodes in Tc1 animals. Conversely, major sleep disturbances are not found in Ts1Cje (Duchon *et al.* 2011), although they do show a delay in sleep rebound (Colas *et al.* 2008). These findings have prompted this group to make some assumptions on the contribution of loci to the sleep disturbance phenotype, suggesting that they should not be triplicated in Ts1Cje. In particular, they noted that APP transgenic mice show consistent sleep disturbances in multiple studies (Colas *et al.* 2004). However, recent findings in Tc1 mice that the final coding exon of APP is rearranged with no human APP protein detectable would argue that loci other than APP contribute to the sleep phenotypes in Down syndrome mutant models (Reinholdt *et al.* 2011).

Although not fully investigated in Tc1 mice, data from human studies and from other mouse models would suggest that rest/activity and rhythm disturbances may arise as a consequence of either generalized synaptic deficits or disturbances in particular brain circuitries. MRI scans of individual Down syndrome patients have recorded numerous brain anomalies including changes in size of cerebellum, frontal, temporal and occipital cortical lobes and hippocampus (Roubertoux & Kerdelhue 2006), while alterations in cortical lamination, dendritic branching and numbers of synapses have also been recorded (Roizen & Patterson 2003). Aside from these general structural deficits, disturbances in cholinergic (Roizen & Patterson 2003) and serotonergic function (Seidl *et al.* 1999) identified in Down syndrome patients would seem most likely to affect activity/sleep parameters. More specifically, deterioration in cholinergic basal forebrain neuronal function may be causative. Most data on neural correlates from mouse models have come from the Ts65Dn line. In line with the human MRI data, dendritic spine density is lower in hippocampus (Belichenko *et al.* 2004) and cortical pyramidal cells of environmentally enriched mutant animals (Dierssen *et al.* 2003). Disturbances in many neurochemical circuits are also evident in this model. Although there is no consensus as to when disturbances in particular circuits contribute to the numerous behavioural phenotypes (Granholm *et al.* 2000; Hunter *et al.* 2004; Seidl *et al.* 1999; Seo & Isacson 2005), it is possible, for example, that subtle effects in cholinergic neurons from early adulthood may be affecting rest/activity patterns in mouse models. Finally, although neuroendocrine function is disturbed in Down syndrome (Roubertoux & Kerdelhue 2006), there is no specific data from mouse work, suggesting that hypothalamic dysfunction contributes to the rest/activity or sleep fragmentation phenotypes seen in mouse models. Future studies into hypothalamic function in Tc1 mice may help in clarifying its contribution to rest/activity and sleep disturbances.

Sleep disturbances may contribute to cognitive dysfunctions in Down syndrome patients, while individuals with high ratings of sleep disruption have greater difficulties with executive functions (Chen *et al.* 2013). In a study using optogenetics tools to disrupt sleep in mice, sleep fragmentation in itself can impair mouse performance in an object recognition task without affecting the overall amount or intensity of sleep (Rolls *et al.* 2011). In general, poor sleep seems to impair memory consolidation (Brown *et al.* 2012; Stickgold 1998) further exacerbating cognitive impairments in Down syndrome patients and warranting further investigation in mouse models. Tc1 mice display a number of these additional traits with deficits evident in a number of learning and memory paradigms (Morice *et al.* 2008; O'Doherty *et al.* 2005).

The investigation of sleep and rhythm-related disturbances in mouse models of Down syndrome shows consistently abnormal parameters, although the contribution of different loci on Hsa21 remains to be clarified. Nevertheless, the continued use of diverse phenotyping tools in different mouse models will be invaluable in furthering our understanding of sleep disturbances in Down syndrome patients.

## Supporting Information

**Figure S1:** Representative actograms focusing on wheel-running in response to a light pulse. Representative actograms for two Tc1 mice and a wild-type control mouse showing wheel-running activity in response to an acute light pulse. Shaded regions indicate where lights are on. Wheel-running behaviour was quite variable, and no significant differences were found.
