# Supplementary material for: Sleep-like behavior and 24-h rhythm disruption in the Tc1 mouse model of Down syndrome
Source: Genes Brain Behav. 2015 Feb 16;14(2):209–16. doi: 10.1111/gbb.12198 (PMC4409853; doi:10.1111/gbb.12198)

**Supplementary Figure 1.**

**Representative actograms focusing on wheel-running in response to a light pulse.** Representative actograms for two Tc1 mice and a wildtype control mouse showing wheel running activity in response to an acute light pulse. Shaded regions indicate where lights are on. Wheel-running behaviour was quite variable and no significant differences were found.


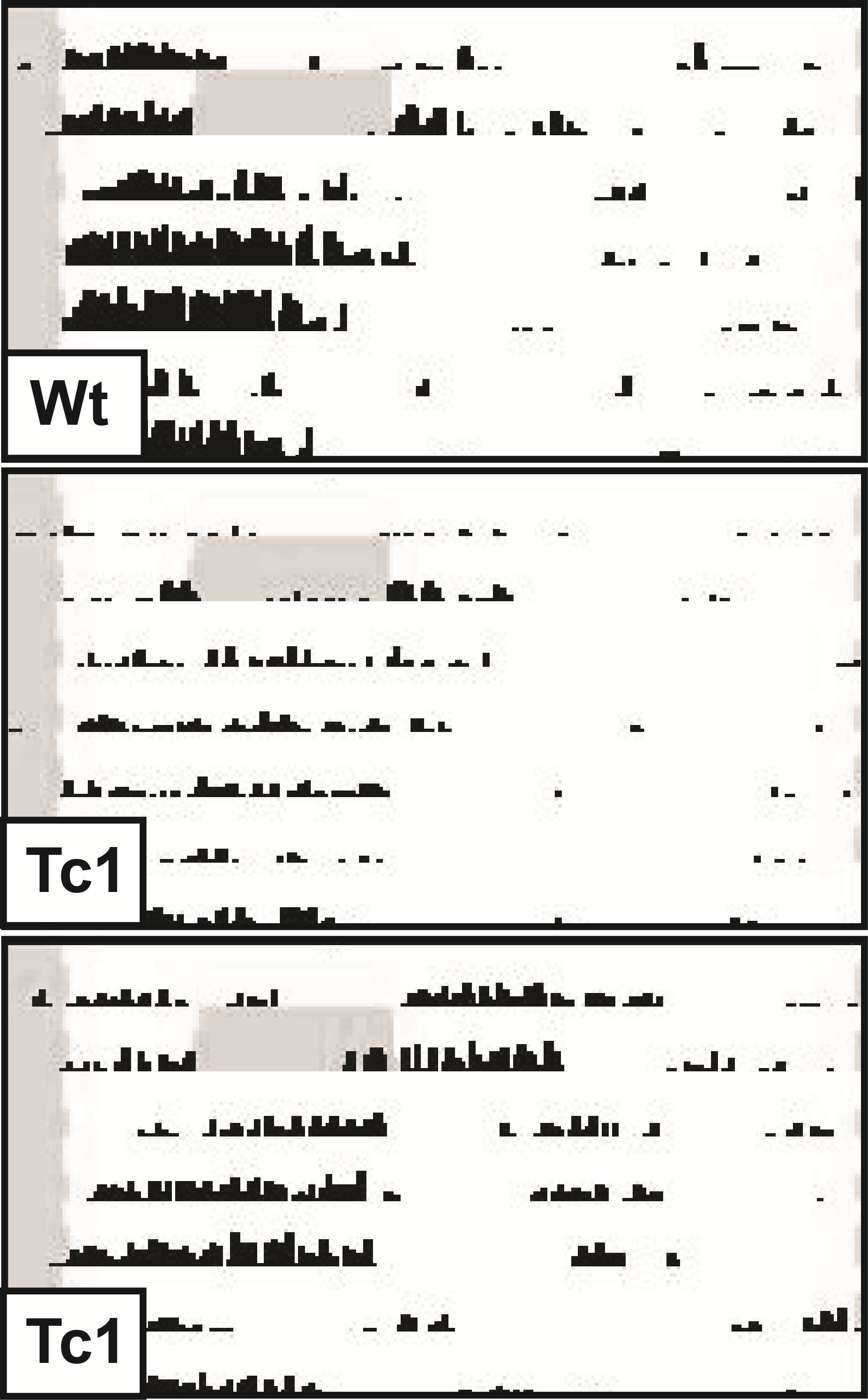

Supplement: Supplementary file 1 — Figure S1: Representative actograms focusing on wheel-running in response to a light pulse. Representative actograms for two Tc1 mice and a wild-type control mouse showing wheel-running activity in response to an acute light pulse. Shaded regions indicate where lights are on. Wheel-running behaviour was quite variable, and no significant differences were found. [file gbb0014-0209-sd1.doc]
